# Micronutrient Deficiencies as Early Signs of Celiac Disease: Exemplified by Two Cases

**DOI:** 10.1155/cjgh/7256866

**Published:** 2025-09-27

**Authors:** Thea Skov Nielsen, Signe Ulfbeck Schovsbo, Allan Linneberg, Line Lund Kårhus

**Affiliations:** ^1^ Center for Clinical Research and Prevention, Copenhagen University Hospital–Bispebjerg and Frederiksberg, Copenhagen, Denmark; ^2^ Department of Clinical Medicine, Faculty of Health and Medical Sciences, University of Copenhagen, Copenhagen, Denmark, ku.dk

**Keywords:** celiac disease, diagnostic delay, micronutrient deficiencies, symptoms

## Abstract

Celiac disease is an important yet often overlooked condition that, when left untreated, can lead to intestinal villous atrophy, resulting in malabsorption and nutritional deficiencies. The clinical presentation of celiac disease varies greatly and is not limited to gastrointestinal symptoms. The diverse clinical picture and nonspecific manifestations can lead to difficulties in the diagnostic process and a delay in diagnosis. In this article, we discuss two cases in which the diagnosis was established—but very late. The first case presents a woman who experienced symptoms for more than 10 years and was initially suspected of having cancer before finally receiving the correct diagnosis of celiac disease. The second case describes a former general practitioner who, at the age of 55 years, underwent transglutaminase antibody testing for celiac disease and only then realized that he could feel differently. Both cases presented early signs of micronutrient deficiencies and/or weight loss, despite maintaining an adequate dietary energy intake. These cases illustrate the challenges with diagnostic delay.

## 1. Introduction

Previously, celiac disease was considered a rare childhood disease. However, today, we know that it is a systemic disease that can develop at any age. It affects about 1% of the population [1]. Celiac disease is a lifelong disease caused by an abnormal immune response in genetically predisposed individuals, triggered by intake of the gluten protein found in wheat, rye, and barley [2]. The only possible treatment for celiac disease is a lifelong gluten‐free diet.

Like other autoimmune diseases, celiac disease has a strong human leukocyte antigen (HLA) association. HLA DQ2 and HLA DQ8 are primary genetic factors for celiac disease, and the HLA‐mediated presentation is an essential part of its pathogenesis. Gluten peptides are deamidated by the tissue transglutaminase (TTG) enzyme and are presented through HLA DQ2 and HLA DQ8 to CD4+ T cells, which lead to the development of celiac disease [2, 3]. In our population‐based study, we found that 48% of the Danes were HLA DQ2 or DQ8 positive and potentially at risk of developing celiac disease [4].

The abnormal immune response triggered by gluten intake causes inflammation of the small intestine mucosa and villous atrophy. These changes can be seen in duodenum biopsies, and gastroscopy with biopsies is a diagnostic criterion in adults, in most cases [5]. The mucosa mostly normalizes after the adoption of a gluten‐free diet [6]. The villous atrophy and inflammation can lead to loss of absorptive surfaces and subsequent nutrient malabsorption [7]. This frequently results in weight loss or difficulties to maintain normal weight, as well as micronutrient deficiencies [8]. Vitamin D is one example, with evidence suggesting a bidirectional relationship. Celiac disease may cause vitamin D malabsorption, while low vitamin D level may impair immune function and potentially worsen disease progression [9]. Iron deficiency, with or without anemia, is another frequent complication, and lack of response to oral iron supplementation is considered a strong indicator to suspect the disease [10]. Other common deficiencies include ferritin, calcium, and vitamin B, which are commonly observed at the time of diagnosis [11].

Although celiac disease primarily affects the small intestine, clinically, celiac disease has both gastrointestinal and extraintestinal symptoms [1]. Because of malnutrition, patients may develop complications such as osteopenia, iron deficiency anemia, headaches, and fatigue [12]. Therefore, celiac disease can be a clinical and diagnostic challenge. A Swedish study has shown that the average time for diagnosing celiac disease is 10 years from the first symptom and 6 years from the first doctor’s visit [13], and we also found marked diagnostic delay among Danish patients with celiac disease [14].

There are several celiac disease‐specific antibodies for diagnostic use. The Danish guidelines [5] recommend measuring TTG‐IgA combined with either total IgA (IgA deficiency is more frequent in individuals with celiac disease) or IgG anti‐deamidated gliadin peptide (DGP). It is important to note that patients need to eat gluten for the antibody test to be positive, as the antibodies often normalize on a gluten‐free diet. If the patient is on a gluten‐free diet, a gluten provocation of at least 4 weeks should be considered [5]. If the antibody test results are positive, the patient should be referred for a gastroscopy to perform duodenal biopsies. Furthermore, it is recommended that all patients with celiac disease are offered dietary guidance from a dietitian, and one should consider a DEXA scan [15].

Even today, despite the attention and new diagnostic markers, many cases of celiac disease remain undiagnosed [1, 16, 17]. We have shown that celiac disease is also significantly underdiagnosed in Denmark. By screening, we found that the prevalence of biopsy‐verified screening‐detected celiac disease was up to 10 times higher than the known incidence of diagnosed celiac disease in the National Patient Register [18]. Additionally, we discovered that many cases of celiac disease could not be found based on the classic gastrointestinal symptoms [19], which have also been found in Sweden [20]. Furthermore, the evaluation of the screening illustrated that some cases who, prior to the screening, had not registered symptoms in the questionnaire and who described themselves as being in good health, experienced an improvement in, e.g., fatigue, headaches, and bloating on a gluten‐free diet. This finding has also been shown in other studies, which indicates that some patients get used to the symptoms and do not realize they have them until they are relieved [21, 22].

Awareness of the disease and its possible symptoms and relatively low‐threshold tests (TTG antibody blood tests in patients suspected of having celiac disease) will decrease the prolonged diagnostic process and avoid many unnecessary years of symptoms and burden of disease. We here present two cases with significant diagnostic delay. The cases highlight weight loss and/or micronutrient deficiencies as early signs of celiac disease.

## 2. Case Presentation

### 2.1. Case 1: A Woman With Severe Fatigue for Over Ten Years Prior to Celiac Disease Diagnosis

In 2009, the patient experienced fatigue and exhaustion for the first time. Therefore, after some time, she contacted her general practitioner (GP). The patient initially attributed her symptoms to stress, as she experienced severe fatigue and widespread body pain. The GP performed blood tests for iron, vitamins, and metabolism. The tests showed vitamin D and iron deficiency, and she was prescribed vitamin D and iron supplements. She agreed to annual blood test checkups. Among other things, they discussed workload, and the patient was told that “… everyone is tired sometimes.” Unfortunately, the patient took it very literally and later acknowledged that she neglected periods of fatigue and exhaustion.

Blood tests results in October 2012 showed low levels of ferritin (14 μg/L) and elevated MVC (98 fL). Otherwise, the blood test results were normal as were hemoglobin (8.5 mmol/L) and iron (16 μmol/L). The 25‐Hydroxy‐Vitamin D (D3 and D2) was 51 nmol/L. In 2014, blood test results showed low levels of ferritin (12 μg/L) and iron (7 μmol/L), as well as a folate deficiency (5.6 nmol/L). She was informed that it is “completely normal” for a woman to have low iron and folate levels, and she was prescribed a folic acid supplement. In 2014, she underwent surgery to remove two benign nodules in glandula parotitis and glandula thyroidea. Metabolism was not affected, and she was discharged from the otorhinolaryngology department.

During this time, the patient experienced considerable weight loss. In 2009, she weighed around 60 kg but then lost weight gradually. In 2014, she weighed 55 kg, 54 kg in 2017, then 51 kg in 2018, and 47 kg in January 2020. The weight loss happened even though she was eating as much as she could. The patient is 164 cm and changed from having a body mass index (BMI) of 22.3 kg/m^2^ in 2009 to a BMI of 17.5 kg/m^2^ in 2020. During this time, she had blood tests taken over 20 times.

She suffered from several infections, and despite getting 10 hours of sleep per night, she was constantly exhausted. She explained, “I had to have 10 hours of sleep per night. Even when I got that, I had to drag myself out of bed and to work.” In January 2020, her GP ordered a series of blood tests (Table 1), and the patient was referred to the department of surgical gastroenterology due to weight loss and suspicion of cancer.

**Table 1:** Blood test results at referral, January 2020 (Case 1).

*Metabolism*	
Cholesterol HDL	1.3 mmol/L
Cholesterol LDL	2.1 mmol/L
Cholesterol	4.1 mmol/L
Triglyceride	1.6 mmol/L
Glucose, average (from HbA1c)	5.9 mmol/L
Hemoglobin A1c (IFCC)	35 mmol/mol
*Endocrinology*	
Thyrotropin (TSH)	4.0 × 10^−3^ IU/L
*Immunology*	
Immunoglobulin A	3.5 g/L
*Autoantibodies*	
Transglutaminase‐IgA (p.d.e.)	1416 (↑)
*Urine and feces*	
Calprotectin	515 × 10^−6^ (↑)
*Hemostasis*	
Coagulation factor II + VII + X (INR)	1.0
*Organ marker*	
Alanine transaminase (ALAT)	32 U/L
Amylase, pancreas type	34 U/L
Alkaline phosphatase	125 U/L (↑)
Bilirubin	< 3 μmol/L (↓)
Gamma‐glutamyl transferase	10 U/L
*Hematology*	
Basophilocytes	0.05 × 10^9^/L
Eosinophilocytes	0.14 × 10^9^/L
Erythrocytes, vol.fr.	0.39
Erythrocytes	4.06 × 10^12^/L
Erythrocyte volume (average) (MCV)	96 fL
Ferritin	7 μg/L (↓)
Folate	3.4 nmol/L (↓)
Iron	3 μmol/L (↓)
Leukocytes	4.3 × 10^9^/L
Lymphocytes	1.27 × 10^9^/L (↓)
Metamyelo. + myelo. + promyelocytes	0.01 × 10^9^/L
Monocytes	0.48 × 10^9^/L
Neutrophilocytes	2.38 × 10^9^/L
Reticulocytes	41 × 10^9^/L
Transferrin saturation	0.04 (↓)
Transferrin	39 μmol/L
Thrombocytes	256 × 10^9^/L
Vitamin B12	387 pmol/L
*Fluid and electrolyte balance*	
Albumin	34 g/L (↓)
Calcium (corrected albumin)	2.42 mmol/L
Calcium	2.34 mmol/L
eGFR/1.73 m^2^ (CKD‐EPI)	> 90 mL/min
Kalium	5.5 mmol/L (↑)
Creatinine	63 μmol/L
Natrium	140 mmol/L

She was also referred for an abdominal ultrasound, apparently due to obstipation. The ultrasound did not show anything abnormal. She was not examined at the hospital concerning suspicion of cancer. However, she was informed that they suspected celiac disease, and she was instead referred for a gastroenterological assessment. Gastroscopy with biopsy was performed due to elevated TTG‐IgA (1416 units/L). Macroscopic observation of the gastroscopy showed obliterated villi and edematous mucosa in her duodenum. The conclusion of the microscopy from the duodenum biopsy was moderate to severe crypt hyperplastic villus atrophy and intraepithelial lymphocytosis compatible with celiac disease.

The patient was finally diagnosed with celiac disease, at the age of 48 years. From March 2020, she started eating a gluten‐free diet. After three weeks, she noticed improvement. She quickly gained weight (6 kg in June and 10 kg in December), fatigue ceased, and blood test results were normalized; in May 2020, ferritin was 116 μg/L, folate was > 45.4 nmol/L, and iron was 22 μmol/L. TTG‐IgA was 59 units/L, 26 units/L, and normalized at 13 units/L in May 2020, September 2020, and January 2021, respectively. A DEXA scan was performed in relation to the diagnosis, which revealed osteoporosis (*T*‐score: −2.6).

After starting a gluten‐free diet, the patient discovered many symptoms retrospectively as they disappeared while eating a gluten‐free diet. She used to suffer of muscle aches and cramps. She had very dry skin and a tendency to experience a flicker in front of her eyes, which also disappeared. She has never had abdominal symptoms except for periodically being constipated at the end of the diagnostic process, which the patient believes was due to the iron supplements. Looking back, she was always very hungry and could eat whatever she wanted, but now she gains weight when she eats more. The patient’s primary symptom in this case was fatigue. After receiving the diagnosis celiac disease and treatment with gluten‐free diet, she has regained energy and can now handle much more activity in her daily life.

### 2.2. Case 2: A Former GP, Who Diagnosed Himself With Celiac Disease

Case 2 is a man, who had a normal childhood and upbringing—no abdominal discomfort. The only thing he noticed was that he was hungrier than other people. He experienced intense hunger. He had to eat every 2 hours, and if he did not eat, he became very irritable and affected. When biking, he experienced total muscle weakness in his legs, and if lunch was postponed, he became irritable and found it difficult to remember what he had just experienced. He was active growing up, playing handball, and biked in his adult life. According to himself, everything was okay as long as he ate enough food; however, compared with others, he experienced that he was “low in blood sugar” very quickly while exercising.

He could eat as much as he wanted without gaining weight. He was 187 cm and 75 kg (BMI: 21 kg/m^2^). In December 2004, he read the article: “Celiac disease—a disease that needs be found in general practice” [23]. It was about celiac disease. It stated that not everyone has classic symptoms and informed about TTG antibody testing. He tested himself for celiac disease utilizing a TTG antibody blood test because of this article. The results came back with elevated levels of celiac disease antibodies, and he was referred for a gastroscopy and duodenum biopsies. He was diagnosed with celiac disease at the age of 55 years.

When diagnosed, all his blood test results were normal except for the elevated TTG (in February 2005: 1049 units/L) and slightly low vitamin D (vitamin D2 + D3 was 36 nmol/L in February 2005). The conclusion of the duodenum biopsies from March 2005 was “small intestinal mucosa with crypt hypertrophic villous atrophy compatible with celiac disease.”

After initiation of a gluten‐free diet, his hunger disappeared, and he no longer felt sick if more than 2 hours passed between meals. He quickly gained 10 kg. Furthermore, he noticed that his nails and beard grew, and his hair got stronger. TTG normalized after starting a gluten‐free diet; by September 2005, it was 67 units/L, and for the last several years, it has been < 7 U/mL. Vitamin D normalized as well; in June 2005, it was 86 nmol/L. In November 2006, approximately 1.5 years after the diagnosis, a control gastroscopy with new biopsies was performed, which showed “duodenal mucosa with a light crypt hypertrophy and chronic inflammation compatible with the treatment effects of a celiac disease‐altered mucosa” (Table 2).

**Table 2:** Clinical characteristics and follow‐up after initiation of a gluten‐free diet (Case 2).

	At diagnosis	After gluten‐free diet
Body mass index	21 kg/m^2^	24.3 kg/m^2^
Transglutaminase‐IgA	1049 units/L	67 units/L (September 2005) and later normalized (< 7 U/mL)
Vitamin D2 + D3	36 nmol/L	86 nmol/L
Biopsies	Villous atrophy with crypt hypertrophy (compatible with celiac disease)	Mild crypt hypertrophy with chronic inflammation (consistent with treated celiac mucosa)

The patient, in this case, was a GP in a shared practice. He experienced that, after his own diagnosis, they had more focus on celiac disease within their practice, resulting in more diagnoses of celiac disease. His experience from talking to other colleagues was that their knowledge of celiac disease was limited, and they did not perform celiac disease antibody tests. His advice to others is that celiac disease needs to be ruled out in many cases, e.g., patients with fatigue or other unspecific symptoms.

## 3. Discussion

Celiac disease is easily overlooked in the clinic because symptoms and clinical findings vary greatly, and classic symptoms from the gastrointestinal system are often minimal or completely absent, as illustrated by these two cases. Even though celiac disease is a systemic disease that can cause different micronutrient deficiencies [24], resulting in different sequelae, many still see celiac disease solely as a gastrointestinal disease, resulting in late suspicion of celiac disease in the diagnostic process, as illustrated above. It is also worth noting that the patients believed that some of their symptoms were normal until they started a gluten‐free diet and realized they could feel different [22]. Therefore, it is important to consider celiac disease if the patient is unwell with unspecific symptoms or blood tests show deficiencies. Awareness of the disease, and relatively low‐threshold testing for celiac disease, can reduce the prolonged diagnostic process and avoid many unnecessary years of symptoms and burden of disease. Early recognition is particularly important, as many deficiencies are expected to improve once the disease is controlled with a gluten‐free diet. However, a delayed diagnosis often means that deficiencies have persisted for years, which may increase the risk of long‐term complications and make supplementation necessary, especially in the period shortly after diagnosis or in individuals with ongoing active disease [8]. In both cases, weight gain was observed after initiation of a gluten‐free diet, likely reflecting villous recovery and improved absorptive capacity of the small intestine [25].

## 4. Conclusions

In conclusion, these cases highlight the significance of recognizing celiac disease as a systemic condition with diverse clinical manifestations, not solely limited to gastrointestinal symptoms. The presented cases underline the diagnostic challenges and potential delays in identifying celiac disease, emphasizing the need for increased awareness among healthcare professionals. Additionally, the study exemplifies that early sign of celiac disease, such as malabsorption, weight loss (despite sufficient dietary energy intake), and nutritional deficiencies, frequently manifest even in the absence of typical intestinal symptoms. Underdiagnosis and diagnostic delay in celiac disease may cause health complications and is a burden for both the patient and society. Awareness of celiac disease in the clinic can reduce prolonged diagnostic process.

## Consent

Informed consent was obtained from all subjects involved in the study.

## Disclosure

All authors have read and agreed to the published version of the manuscript.

## Conflicts of Interest

The authors declare no conflicts of interest.

## Author Contributions

Conceptualization: L.L.K. and A.L.; collection of clinical data and writing the first draft in Danish: L.L.K.; preparation of the final version of the manuscript: T.S.N.; review and supervision: L.L.K., A.L., and S.U.S.

## Funding

This research received no external funding.

## Data Availability

Data sharing is not applicable to this article as no datasets were generated or analyzed during the current study.
